# Ultrasound-Assisted Extraction of Methylxanthines from Cocoa Bean Shells Using Glycerol–Urea Natural Deep Eutectic Solvent (NADES): Optimization and Greenness Assessment

**DOI:** 10.1021/acsomega.5c05241

**Published:** 2025-09-16

**Authors:** Luciana L Nascimento, Paulo N. A. dos Santos, Luciano Albuquerque, Bruna L. de M. Pita, Lorena S. de Almeida, Maira L. de Oliveira, Camila de A. Moreira, Fernanda R. P. Teixeira, Elina B. Caramão, Fabio de S. Dias, Alini T. Fricks

**Affiliations:** † Programa de Pós-Graduação em Ciência de Alimentos, 28111 Universidade Federal da Bahia, Salvador, BA 40170-115, Brazil; ‡ Rede Nordeste de Biotecnologia, 74391 Universidade Federal de Sergipe, São Cristóvão, SE 49107-230, Brazil; § Departamento de Análises Bromatológicas, Faculdade de Farmácia, Universidade Federal da Bahia, Salvador, BA 40170-115, Brazil; ∥ Programa de Pós-Graduação em Química, Universidade Federal da Bahia, Salvador, BA 40170-115, Brazil

## Abstract

This study reports the optimization of ultrasound-assisted extraction of theobromine and caffeine from cocoa bean shells using glycerol–urea natural deep eutectic solvents. The solvents were characterized by rheological analysis and infrared spectroscopy. A Doehlert design was applied to optimize sonication time (60–300 s) and water content (30–70% v/v) in the glycerol–urea natural deep eutectic solvents formulations. Theobromine and caffeine concentrations, quantified by high-performance liquid chromatography, were used as response variables, and simultaneous optimization was performed using the desirability function. Under optimized conditions (45.5% water and 4 min sonication), the method yielded 29.18 ± 0.07 mg g^–1^ DM of theobromine, 2.46 ± 0.04 mg g^–1^ DM of caffeine, and 91.79 ± 3.77 mg GAE g^–1^ DM of total phenolics. Antioxidant capacity was also high, with values of 230.65 ± 5.41 mg TE g^–1^ DM (ABTS) and 74.34 ± 3.84 mg TE g^–1^ DM (DPPH). The high-performance liquid chromatography method demonstrated good linearity, precision, accuracy, and detection limits. Greenness assessment indicated low environmental impact (AGREE = 0.55/1.00; AGREEprep = 0.67/1.00), confirming the method’s alignment with green chemistry principles. Overall, the proposed approach represents a sustainable and efficient alternative for the recovery of bioactive compounds from cocoa processing residues. The workflow uses inexpensive, food-relevant components, short sonication times, and moderate temperatures, supporting transferability to continuous ultrasound reactors. The resulting methylxanthine-rich extract is suitable for downstream formulation of food, beverage, or nutraceutical ingredients after routine polishing/dilution steps. The solvent can be prepared from bulk commodities and reused, aligning with circular economy strategies for cocoa processing.

## Graphical abstract

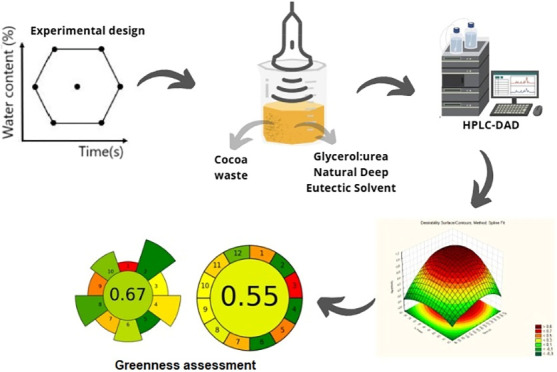

## 1 Introduction

Food waste is a critical global challenge at the intersection of food science and sustainability. Each year, approximately one-third of all food produced for human consumption is lost or wasted,^1^ contributing to an estimated 8–10% of global greenhouse gas emissions.^2^ In response, international initiatives emphasize waste reduction and valorization through circular food strategies. For instance, the United Nations Sustainable Development Goal 12.3 aims to halve per capita global food waste by 2030.^3^ Converting agri-food industry (AFI) byproducts into value-added products is a key strategy to meet these goals, transforming waste into valuable resources and mitigating environmental impacts.^4^ Therefore, innovative and green extraction strategies are needed to fully harness this potential.

Cocoa bean shells (CBS), the primary byproduct of cocoa processing, are typically discarded or used in low-value applications such as animal feed or fertilizer.^5,6^ However, CBS is an underutilized resource with significant potential in the food industry due to its high content of bioactive compounds, including methylxanthines (such as theobromine and caffeine) and polyphenols. These compounds possess antioxidant, stimulant, and potential health-promoting properties, making CBS a valuable ingredient for functional food formulations.^7,8^ Sustainable extraction techniques are required to efficiently harness these bioactive compounds while maintaining the principles of green chemistry. Recently, several nonconventional methods such as microwave-assisted extraction,^8^ ultrasound-assisted extraction (UAE),^9,10^ pressurized liquid extraction,^11^ and energized dispersive guided extraction,^12^ among others, have been explored for the efficient recovery of these valuable compounds.

UAE has emerged as an effective green technology for recovering bioactive compounds from food industry byproducts.^13^ The physical effects of acoustic cavitation enhance mass transfer and promote the breakdown of plant cell structures, facilitating compound release.^9,14,15^ Compared to conventional solvent extraction methods, UAE offers advantages such as reduced extraction time, lower solvent consumption, and improved efficiency.^9,16^ In analytical sample preparation, UAE has been successfully employed for the extraction of phenolics, alkaloids, flavonoids, and other bioactive compounds from a variety of complex plant and food matrices prior to chromatographic or spectrophotometric analysis. This technique improves analyte recovery, reduces matrix interferences, and contributes to method miniaturization and automation in green analytical chemistry workflows.^10,13,15^ When combined with environmentally friendly solvents, UAE aligns with sustainable food processing goals and supports the transition to greener extraction methods.^13^

Natural deep eutectic solvents (NADES) have gained attention as promising alternatives to traditional organic solvents in bioactive compound extraction. These solvents, composed of naturally derived hydrogen bond donors (HBD) and acceptors (HBA), exhibit tunable physicochemical properties, low volatility, and biodegradability.^13,17^ NADES are particularly advantageous for food applications as they can enhance the solubility of bioactive compounds and provide a safer, more sustainable extraction medium compared to conventional solvents.^18^ Their combination with UAE offers a novel approach to improving the efficiency and environmental impact of bioactive compound recovery from food waste streams. Glycerol-based NADES have previously shown strong performance in botanical extractions, and DES/NADES media are particularly effective for alkaloids such as methylxanthines, which exhibit favorable solubility and stability in such systems.^19–21^

Despite the promising potential of CBS for food applications, efficient and sustainable extraction methods are essential for maximizing bioactive compound recovery. Optimization plays a crucial role in ensuring high yields while maintaining environmentally friendly conditions. In this study, ultrasound-assisted extraction with natural deep eutectic solvents was optimized to enhance the recovery of theobromine and caffeine under green chemistry principles. This approach aligns with sustainable food processing trends and contributes to the valorization of food industry byproducts. Additionally, a reversed-phase high-performance liquid chromatography coupled with diode array detection (HPLC-DAD) method was developed and validated for quantification of theobromine and caffeine. The greenness of the proposed extraction method was assessed using AGREE and AGREPrep metric tools to ensure its alignment with sustainability standards.

## 2 Methods

### 2.1 Plant Material and Chemicals

Cocoa bean shells (CBS) were obtained from an organic chocolate factory in Salvador, Bahia, Brazil (geographic coordinates: latitude −12° 54′ 13.6728″, longitude −38° 26′ 34.3926″). The CBS was ground using a standard laboratory mill and sieved to obtain particles smaller than 0.841 mm (20 mesh).

### 2.2 NADES Preparation

Glycerol-urea NADES was prepared in a 1:1 molar ratio, following previously reported protocols.^19–21^ During preliminary tests, other molar ratios (such as 2:1 and 1:2) were evaluated. However, these alternative formulations resulted in phase instability, including the formation of precipitates and incomplete homogenization. The 1:1 ratio exhibited the most favorable physicochemical characteristics, producing a clear and stable mixture, and was therefore selected for further experiments.The hydrogen bond acceptor (glycerol) and hydrogen bond donor (urea) were mixed and stirred in a flask at 80 °C until a homogeneous, transparent, and colorless liquid was obtained.^22^

To evaluate the effect of water content on the extraction process, NADES solutions with varying water concentrations were prepared by diluting the original NADES with deionized water. The resulting solutions contained 0% (NADES0), 30% (NADES30), 50% (NADES50), and 70% (NADES70) water (v/v).

### 2.3 Determination of Physical Properties of NADES

#### 2.3.1 Fourier-Transform Infrared Spectroscopy (FTIR) Analysis

The main functional groups present in the NADES were identified using FTIR spectroscopy with an attenuated total reflectance (ATR) technique. The analysis was conducted using a Shimadzu IR Prestige 21 spectrometer, operating within a spectral range of 600–4000 cm^–1^ with a resolution of 32 scans per sample.^23^

#### 2.3.2 Apparent Viscosity

Rheological measurements of NADES at different dilutions (NADES0, NADES30, NADES50, NADES70) were conducted using a rheometer with concentric cylinders (Haake Rheotest 2.1) with a sample volume of 5 mL. The apparent viscosity was determined at 25 °C as a function of shear rate, ranging from 25 to 1000 s^–1^.

The rheological behavior of NADES was analyzed using the Power Law model,^24^ which describes the nonlinear variation of shear stress as a function of shear rate, as represented by eq 1

1 $$\tau =K\cdot \gamma^{n}$$

where τ is the shear stress (Pa), γ is the shear rate (s^–1^), *K* is the consistency index of the fluid (Pa·s*^n^*), and *n* is the flow behavior index of the fluid. The apparent viscosity of the fluid is defined as the relationship between shear stress and shear rate, as described by eq 2

2 $$\mu =\frac{\tau }{\gamma }=K\cdot \gamma^{n-1}$$

where *n* < 1. The flow behavior index (*n*) characterizes the fluid type: *n* < 1 indicates a pseudoplastic fluid, *n* > 1, indicates a dilatant, and *n* = 1, indicates Newtonian fluid.

#### 2.3.3 pH

The pH was measured with a pH meter (Orion Star A214, Thermo Fisher Scientific, Waltham, MA, USA).

### 2.4 Ultrasound-Assisted Extraction

A total of 100 mg of cocoa bean shell (CBS) was thoroughly mixed with 3 mL of NADES in a flask. The flask was then placed in an ultrasonic probe system (Ecosonics QR850, 20 kHz, 850 W, Brazil) and processed at 25 ± 2 °C. The extraction was performed at a power of 255 W under conditions defined by the Doehlert design (Table 1). After extraction, the samples were centrifuged (Eppendorf 5453 minispin, Germany) at 4400 rpm for 10 min. The resulting extracts were transferred to amber flasks and stored at −8 °C for further analysis.

**Table 1:** Main Characteristic Band Assignments of the FTIR for Urea, Glycerol and the Proposed NADES

urea	glycerol	NADES0	NADES30	NADES50	NADES70	assignment^a^	observation
3254		3297	3297	3297	3297	ν_as_NH_2_	shift of +43 cm^–1^ in NADES
3429		3394	3394	3394	3394	ν_as_NH_2_	shift of –35 cm^–1^ in NADES
1601		1601	1601	1601	1601	δ NH2	increased intensity in NADES
1100		1100	1100	1100	1100	νCN	increased intensity in NADES
	3478–3111	3494–3107	3500–3106	3500–3106	3500–3105	νH_2_O	broadening
	930	930	930	930	930	δOH	increased intensity in NADES

a ν = stretching; δ = bending; as = asymmetric vibration; s = symmetric vibration.

### 2.5 Optimization Strategy

The optimal UAE performance was determined by applying the Doehlert design to the independent variables: sonication time (min) and water percentage of NADES. This design required nine experiments, which were performed in a random order to avoid any systematic error. The response of interest, called Overall Desirability (D), consisted of a combination of responses obtained using HPLC-DAD, namely theobromine (mg g^–1^ DM) and caffeine (mg g^–1^ DM). Table 1 shows the factors, levels, and experimental design of the Doehlert matrix with the respective responses for each run. All experimental data were processed using Statistica software.^25^

### 2.6 Liquid Chromatographic Analysis

Methylxanthines present in the extracts obtained using UAE (Table 1) were identified and quantified using high-performance liquid chromatography. Samples were analyzed using an HPLC model LC20AD (Shimadzu, Tokyo, Japan) system consisting of a ternary pump, online degasser and automatic sampler and diode array detector (DAD). An RP-C18 column, 4.6 × 150 mm^2^ with porosity of 5.0 μm (Phenomenex, Torrance, CA, USA), and a guard column (4.6 mm ID × 12.5 mm) were employed. The oven temperature was maintained at 45 °C. The mobile phase was composed of water: acetic acid (99:1) (Solvent A) and acetonitrile (Solvent B) at a flow rate of 1.0 mL min^–1^. An isocratic mode was employed using 80% solvent A. The methylxanthines were detected at 273 nm, and the ultraviolet (UV) spectra of individual peaks were recorded within a range of 200–400 nm. Theobromine and caffeine were identified by matching their retention times against those of standards. Quantification was achieved using the linear calibration curves of standards (Sigma-Aldrich, Germany).

### 2.7 Analytical Performance

The analytical performance of HPLC method was evaluated using the following parameters: linearity, limit of detection (LOD), limit of quantification (LOQ), precision, accuracy. The linearity was determined by the external standard method. Calibration curves were constructed by injecting mixed working standard solutions. The linearity was evaluated at eight concentrations (1–100.0 mg L^–1^). The LOD and LOQ were estimated in the current investigation using 3Sd/m and 10Sd/m, respectively, where Sd represents the blank standard deviation and m is slope of calibration curve.^26^ The precision was assessed according to the intraday and interday precisions and was expressed as the relative standard deviation (RSD) at two concentrations (10 mg L^–1^ and 30 mg L^–1^) using standard solutions. The accuracy was evaluated by estimating the relative recovery (RR, %) in relation to each analyte, according to the eq 3

3 $$\mathrm{RR}\;\left(\%\right)=\frac{C_{s}-C_{o}}{C_{\mathrm{add}}}$$

where *C*_s_ is the result of the spiked samples, *C*_o_ is the result of the unspiked samples, and *C*_add_ is the amount of analyte added.

### 2.8 Analytical Greenness Metric Tools

#### 2.8.1 Agree

The AGREE (Analytical GREEnness) metric software^27^ was utilized to evaluate the environmental impact of the analytical methodology. This tool is based on the 12 principles of Green Analytical Chemistry (GAC), which systematically assess various aspects of analytical procedures related to sustainability and safety. Each principle is assessed through a set of quantitative and qualitative criteria, with scores ranging from 0 to 1, where 1 represents the most environmentally friendly approach. These scores are combined into a final AGREE score, which is presented in a circular pictogram. The pictogram visually represents the greenness of each principle using a color-coded scale from red (least green) to green (most sustainable).

#### 2.8.2 AGREEprep

The AGREEprep green metric software^28^ has been used to estimate the greenness of the sample extraction step. This metric tool is based on the 10 principles of green sample preparation.^29^ In the software, each principle is represented by a criterion, which is evaluated and scored from 0 to 1. These scores are weighted and combined to produce an overall score, also ranging from 0 to 1, with 1 representing the ideal, most environmentally friendly approach. Additionally, the score is accompanied by an intuitive color scale from red to green. The software output includes a colorful pictogram that visually represents the assessment of each criterion along with the overall score. The closer the final score is to 1, and the greener it appears, the more environmentally friendly the procedure is considered.

### 2.9 Determination of Total Phenolic Content (TPC)

The phenolic content of the extracts was determined using the methodology of Folin and Ciocalteu,^30^ with some modifications. Briefly, 20 μL of extract was mixed with 500 μL of the Folin reagent. 5 mL of sodium carbonate solution (7% w/v in distilled water) was added to the mixture after 3 min. The reaction was performed in the dark for 2 h. A UV–vis spectrophotometer (FEMTO 800 XI, São Paulo, Brazil) was used to read the samples and standard at 760 nm. Gallic acid was used as a standard, and the results are expressed as milligrams of gallic acid equivalent per gram of dry plant material (mg GAE g^–1^ DM^–1^). For each assay, a NADES-only blank was prepared to match the solvent composition, dilution, and assay conditions of the corresponding sample.

### 2.10 Determination of Antioxidant Activity

#### 2.10.1 DPPH Radical Scavenging Analysis

The DPPH radical scavenging capacity was determined according to Benítez-Correa et al.,^31^ with some modifications. Briefly, 0.1 mL of the adequately diluted sample was mixed with 2.9 mL ethanolic DPPH solution. The reaction was performed at room temperature in the dark for 30 min. A UV–vis spectrophotometer (FEMTO 800 XI, São Paulo, Brazil) was used to obtain the samples and standard readings at 515 nm. Trolox reagent was used as a standard, and the results are expressed as milligrams of Trolox equivalent per gram of dry plant material (mg TE g^–1^ DM). For each assay, a NADES-only blank was prepared to match the solvent composition, dilution, and assay conditions of the corresponding sample.

#### 2.10.2 ABTS Radical Scavenging Analysis

The ABTS free radical decolorization assay was performed according to Mellinas et al.,^8^ with some modifications. The radical monocation of ABTS was generated by the reaction of the ABTS solution (7 mM) with 2.45 mM potassium persulfate at room temperature for 16 h in the dark. The absorbance of ABTS solution was adjusted to 0.70 ± 0.02 with absolute ethanol at 734 nm. 0.15 mL of extract was mixed with 2.85 mL of ABTS solution. A UV–vis spectrophotometer (FEMTO 800 XI, São Paulo, Brazil) was used to obtain the samples and standard readings at 734 nm. Trolox was used as the standard to construct a calibration curve. The results are expressed as milligrams of Trolox equivalent per gram of dry matter (mg TE g^–1^ DM). For each assay, a NADES-only blank was prepared to match the solvent composition, dilution, and assay conditions of the corresponding sample.

## 3 Results and Discussion

### 3.1 NADES Characterization

The extraction of bioactive compounds using NADES can be challenging due to their high viscosity, which limits mass transfer and requires higher energy input to facilitate solvent–solute interactions. To address this limitation, water is often introduced as a cosolvent to reduce viscosity and improve extraction efficiency.^32^ However, excessive water content can disrupt hydrogen bonding between the hydrogen bond donor (HBD) and acceptor (HBA), potentially altering the structural integrity of NADES.^33,34^ Therefore, careful optimization of water content is essential to maintain the balance between viscosity reduction and structural stability.

In this study, NADES were prepared with varying water dilutions (0–70% v/v). FTIR spectroscopy was employed to assess molecular interactions and structural changes indicative of NADES formation. Additionally, the rheological behavior and pH of the prepared NADES formulations were evaluated.

#### 3.1.1 FTIR Analysis

FTIR spectroscopy is a widely used technique for confirming NADES formation by detecting functional group interactions and hydrogen bonding patterns. Shifts in absorption peaks, peak broadening, intensity variations, and the emergence or disappearance of specific bands provide insights into eutectic formation.^35^

Table 1 and Figure 1 presents the characteristic FTIR band assignments for the components of the NADES formulations. In pure glycerol, the O–H stretching vibration appeared as a broad band in the 3478–3111 cm^–1^ region, with a full width at half-maximum (FWHM) of 367 cm^–1^. In NADES0, this band broadened to 387 cm^–1^, suggesting increased hydrogen bonding interactions between hydroxyl groups from glycerol and amine groups from urea.^34,36^ As the water content increased (NADES30, NADES50, NADES70), further broadening was observed, reaching a FWHM of 395 cm^–1^ in NADES70. This progressive increase in bandwidth indicates the formation of an extended hydrogen bonding network due to the incorporation of water molecules.

**Figure 1:**
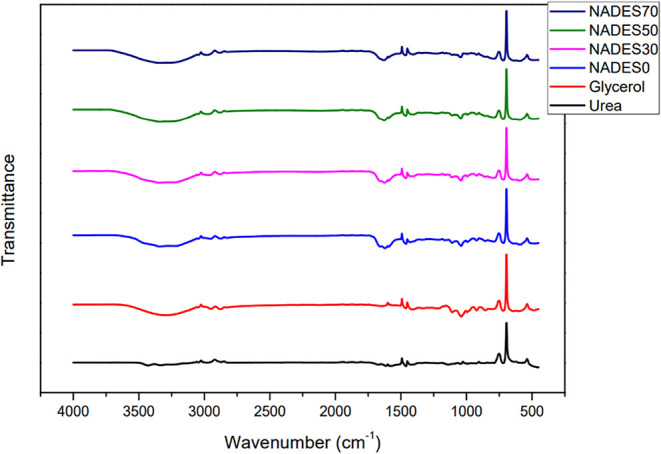
FTIR spectra for urea, glycerol and the proposed NADES.

The symmetric stretching vibration of the −NH_2_ group in urea exhibited a shift of +43 cm^–1^, appearing at 3297 cm^–1^ in all NADES formulations regardless of water content. Similarly, the asymmetric stretching vibration of −NH_2_ was identified at 3394 cm^–1^ across all NADES, corresponding to a shift of −35 cm^–1^ relative to pure urea. These spectral changes indicate strong interactions between amine hydrogens from urea and hydroxyl oxygens from glycerol.

Additionally, all NADES samples showed an increase in band intensity near 1600 cm^–1^, attributed to the bending vibration of the −NH_2_ group (δNH_2_), further supporting the presence of hydrogen bonding. The FTIR assignments of urea’s nitrogen-containing functional groups were consistent with literature reports.^37^ The observed NH···NH, NH···OH, HO···HO, and OH···NH hydrogen bonds confirm the successful formation of the eutectic mixture and its structural stability across water dilutions up to 70%.

#### 3.1.2 Rheological Properties and Apparent Viscosity

Apparent viscosity is a crucial factor influencing the extraction efficiency of bioactive compounds. High viscosity in NADES can hinder mass transfer, leading to inefficient dissolution and prolonged extraction times.^38^ The viscosity of NADES at a constant shear rate of 25 s^–1^ is presented in Table 2.

**Table 2:** Apparent Viscosity (25 °C, Shear Rate of 25 s^–1^) and pH of NADES Formulated at Different % of Water^a^

formulation	apparent viscosity (mPa·s)	pH
NADES0	38.2 ± 0.3^a^	8.53 ± 0.01^a^
NADES30	20.9 ± 0.5^b^	8.49 ± 0.02^b^
NADES50	20.5 ± 0.4^b^	8.37 ± 0.01^c^
NADES70	19.3 ± 0.2^c^	8.31 ± 0.03^d^

a Means followed by different letters, in the same column, differ statistically from each other according to Tukey’s test at a 95% confidence level (*p* < 0.05).

NADES0 exhibited high viscosity (38.2 ± 0.3 mPa·s) due to extensive hydrogen bonding interactions. Introducing water significantly reduced viscosity, with a 43% decrease observed from NADES0 to NADES30. No statistically significant difference was observed between NADES30 and NADES50, while NADES70 exhibited a slightly lower viscosity than both. These results highlight the plasticizing effect of water on NADES, where additional water weakens intermolecular hydrogen bonding, enhancing fluidity.

#### 3.1.3 Rheological Behavior and Flow Properties

The rheological behavior of NADES (Figure 2) was further assessed by analyzing viscosity variation with shear rate. The data fitted well to the Ostwald-de-Waele (Power Law) model, with correlation coefficients (*R* ^2^ > 0.97) indicating an excellent fit. The flow behavior index (*n*) < 1 across all formulations confirmed pseudoplastic (shear-thinning) behavior, where viscosity decreases as shear rate increases.^24^

**Figure 2:**
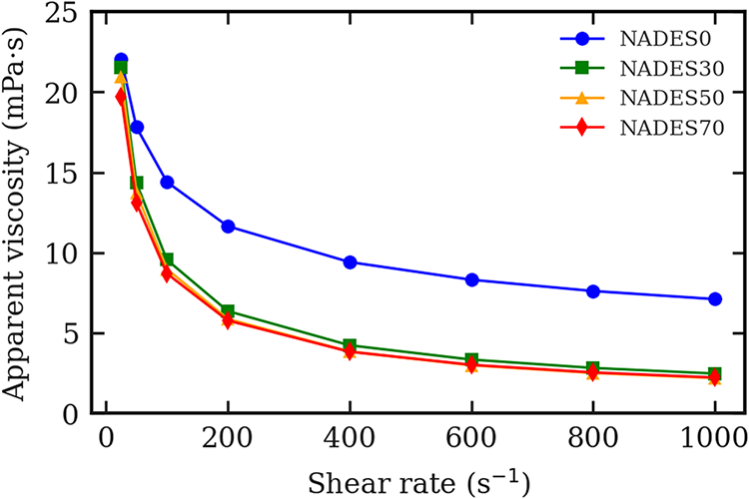
Apparent viscosity as a function of shear rate at 25 °C for natural deep eutectic solvents formulated at different % of water.

The pseudoplastic behavior of the formulated NADES offers important perspectives related to UAE. Cavitation induced by UAE promotes intense micromixing within the fluid, which can temporarily reduce the fluid’s apparent viscosity, making it behave in a less viscous manner during extraction. The shear-thinning characteristics of pseudoplastic fluids, combined with the effects of ultrasonic energy, can facilitate a reduction in viscosity, thereby enhancing extraction efficiency. For instance, Abbas et al.^39^ reported a substantial reduction in the viscosity of vacuum gas oil, characterized as a pseudoplastic fluid, when using ultrasound technology.

#### 3.1.4 pH Analysis of NADES Formulations

In addition to FTIR and rheological data, pH measurements were conducted for all NADES formulations (Table 3). The eutectic mixture (glycerol:urea) exhibited an alkaline pH, which decreased progressively with increasing water content. This trend suggests that water modulates the interactions between the eutectic components, affecting ionization equilibria within the system.

**Table 3:** Coded Values, Real Values, and Respective Responses to the Doehlert Matrix

#	time (s)	water % (v/v)	theobromine (mg g^–1^ DM)	caffeine (mg g^–1^ DM)	overall desirability (*D*)
1 (C)	0 (180)	0 (50)	28.73	2.42	0.83
2 (C)	0 (180)	0 (50)	29.22	2.60	0.98
3 (C)	0 (180)	0 (50)	29.43	2.47	0.91
4	1 (300)	0 (50)	28.84	2.45	0.85
5	0.5 (240)	0.86 (70)	24.17	2.10	0.28
6	–1 (60)	0 (50)	22.60	1.84	0.00
7	–0.5 (120)	–0.86 (30)	27.80	1.95	0.34
8	0.5 (240)	–0.86 (30)	28.80	2.39	0.82
9	–0.5 (120)	0.86 (70)	23.51	1.88	0.09

#### 3.1.5 Selection of NADES

In summary, FTIR analysis indicates the formation and stability of NADES across water dilutions up to 70%. The viscosity and rheological data demonstrated the shear-thinning behavior of NADES, facilitating its application in ultrasound-assisted extraction. Based on these findings, NADES30, NADES50, and NADES70 were selected for further studies on the extraction of bioactive compounds from cocoa bean shells. NADES0 was excluded owing to its high viscosity that hampers efficient extraction.

### 3.2 Optimization of the Ultrasonic-Assisted Extraction

To determine the optimal conditions for sonication time and water content (%) in NADES during extraction, a Doehlert design was employed. The Doehlert design offers an economical and versatile approach to response-surface modeling, providing flexible specification of factor levels. Relative to central composite and Box–Behnken designs, it generally achieves comparable modeling accuracy with fewer experiments.^40^ A multiresponse approach was used to simultaneously optimize theobromine and caffeine content with a mathematical-statistical tool based on a desirability function.

The desirability function, proposed by Derringer and Suich,^41^ is a widely used method for multiresponse optimization. This approach transforms multiple response variables into a single response, referred to as Overall Desirability (*D*). By fitting a mathematical model to *D* values, the behavior of the system can be described, enabling the optimization of experimental conditions.

The first step in obtaining Overall Desirability (*D*) is calculating individual desirabilities (di) for each response variable. Each individual response (yi) is converted into an individual desirability (di) using eq 4

4 $$\mathrm{di}=\frac{\mathrm{yi}-L}{H-L}$$

where *L* and *H* represent the lower and upper observed values for each response variable, ensuring that 0 ≤ di ≤ 1.

The Overall Desirability (*D*) is then computed as the geometric mean of all individual desirabilities (eq 5)­

5 $$D={\left(d1\cdot d2\cdot d3\cdot \cdot \cdot \cdot \mathrm{dk}\right)}^{1/k}$$

where *k* is the number of responses (in this case, 2).

Table 3 presents the coded and actual values of the factors, along with the measured theobromine and caffeine concentrations (mg g^–1^ DM) for each experiment, and the corresponding *D* values computed using eq 4. The predictive performance of the fitted mathematical model was assessed through analysis of variance (ANOVA) at a 95% confidence level. The results indicated that a quadratic model provided the best fit for *D* values.

The model was significant (*p* = 0.0059 < 0.05) and did not show a lack of fit (*p* = 0.4361 > 0.05). Furthermore, values obtained for the coefficient of determination (*R* ^2^) and adjusted coefficient of determination (*R* ^2^ adj) for the model (0.9854 and 0.9610, respectively) indicated a good agreement between the experimental and predicted values.

Figure 3 shows the individual desirability profiles (related to each analyte) and the overall desirability profile indicating the optimum proportions of each component. In addition, the contour plots and response surface are available on Figure 4. The system produced a desirability close to 0.98, value considered as acceptable and excellent.^42^ According to the desirability profile, the optimal extraction conditions for performing simultaneous extractions of the studied analytes were: 45.5% of water and 4 min of sonication. Under optimal conditions, the mean experimental values of theobromine and caffeine were, respectively, 29.18 ± 0.07 mg g^–1^ DM and 2.46 ± 0.04 mg g^–1^ DM (Table 4). The calculated experimental desirability value was 0.91, considered acceptable and excellent, since it is composed of the geometric mean of individual desirabilities for each response under study. Experimental desirability agrees with predicted desirability, attesting to the quality of the obtained model.

**Figure 3:**
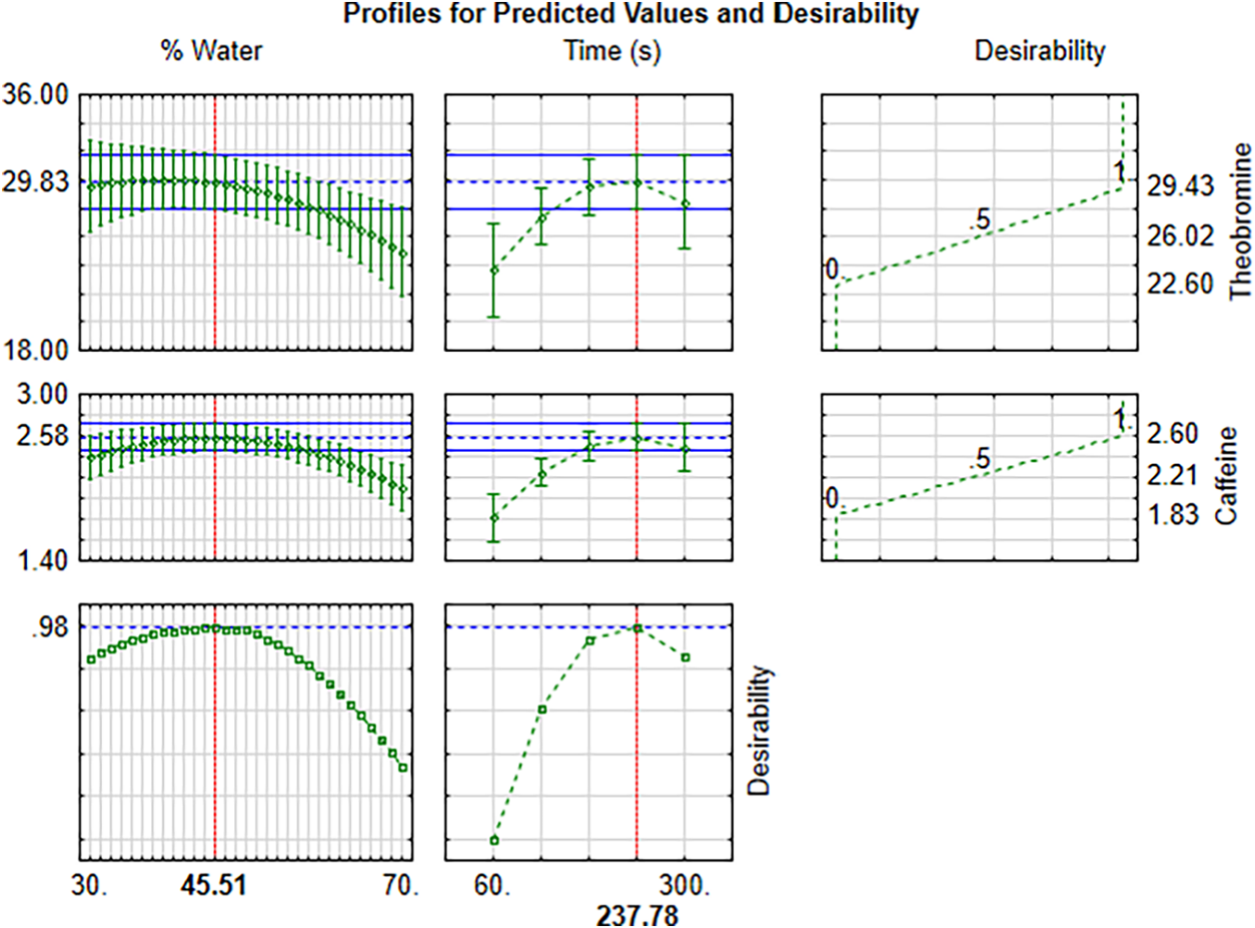
Profiles for predictive values and overall desirability in optimizing the extraction conditions during sonication.

**Figure 4:**
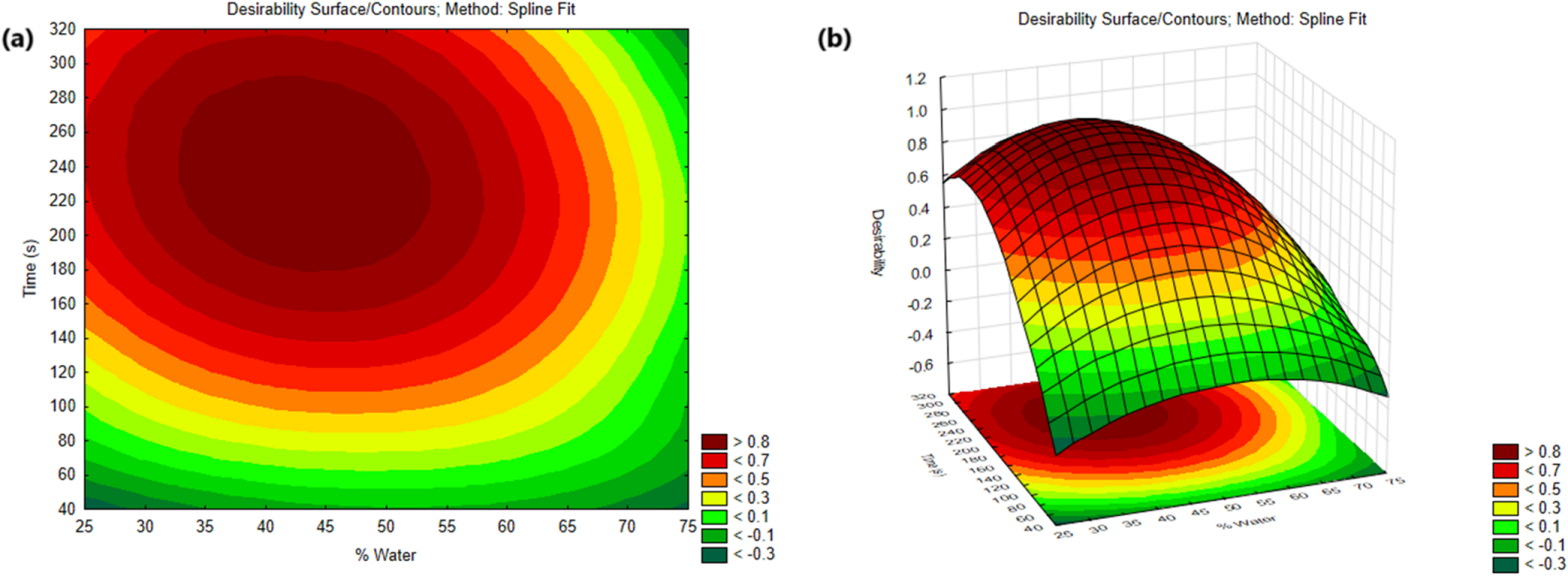
Overall desirability (*D*) from the Doehlert design for co-optimization of theobromine and caffeine extraction using a glycerol–urea NADES: (A) contour and (B) 3D response-surface plots versus sonication time (min) and NADES water content (%, v/v).

**Table 4:** Theobromine, Caffeine, TPC, and Antioxidant Activities (ABTS and DPPH) under Optimal Conditions

solvent	theobromine (mg g^–1^ DM)	caffeine (mg g^–1^ DM)	TPC (mg GAE g^–1^ DM)	ABTS (mg TE g^–1^ DM)	DPPH (mg TE g^–1^ DM)
NADES	29.18 ± 0.07	2.46 ± 0.04	91.79 ± 3.77	230.65 ± 5.41	74.34 ± 3.84
ethanol 40%	18.41 ± 0.15	2.01 ± 0.14	78.17 ± 2.31	112.11 ± 1.19	51.22 ± 1.79

Figure 5 presents a representative chromatogram obtained under optimal extraction conditions using HPLC-DAD. The peaks corresponding to theobromine and caffeine were clearly resolved, with retention times of 3.47 and 4.88 min, respectively. This confirms the selectivity and effectiveness of the analytical method employed for quantification.

**Figure 5:**
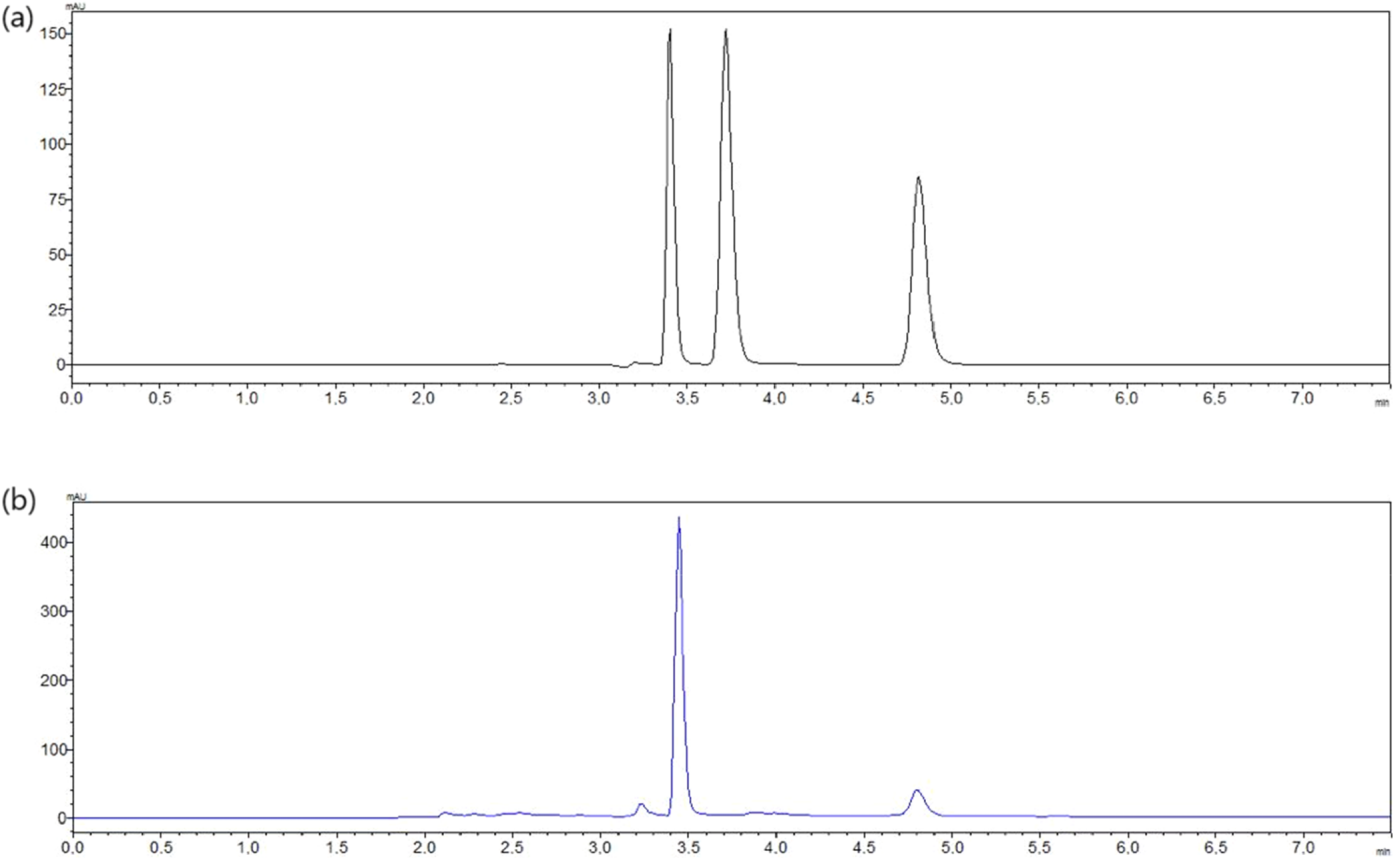
HPLC-DAD chromatograms of (a) standard mixture and (b) cocoa bean shell (CBS) extract obtained under optimal conditions using glycerol–urea NADES. Theobromine and caffeine were detected at retention times of approximately 3.5 and 3.8 min, respectively.

The optimized ultrasound-assisted extraction (UAE) protocol using glycerol–urea NADES developed in this study proved highly effective for recovering methylxanthines from CBS, yielding 29.18 ± 0.07 mg g^–1^ DM of theobromine and 2.46 ± 0.04 mg g^–1^ DM of caffeine. When compared to our previous work using 40% ethanol with the Energized Dispersive Guided Extraction (EDGE) system,^12^ the current method achieved a 45% increase in theobromine content, while caffeine yield was 30% lower, suggesting a selective enhancement in theobromine extraction—likely due to hydrogen bonding and molecular interactions between NADES and theobromine molecules.

Other studies have reported lower or comparable extraction yields using various techniques. Mellinas et al.,^8^ for example, obtained 7.62 mg g^–1^ DM of theobromine via microwave-assisted extraction (MAE) at 97 °C for 5 min. Benítez-Correa et al.^43^ used choline chloride–lactic acid NADES combined with pulsed electric field (PEF) treatment and reported theobromine and caffeine yields ranging from 2.59 to 5.80 mg g^–1^ DM and 0.87 to 2.08 mg g^–1^ DM, respectively. Grillo et al.,^7^ applying UAE and hydrodynamic cavitation, achieved theobromine concentrations between 17 and 32 mg g^–1^ DM and caffeine levels ranging from 1.43 to 1.76 mg g^–1^ DM.

Taken together, these comparisons reinforce the high efficiency and selectivity of the NADES–UAE system, positioning it as a robust and sustainable alternative for methylxanthine extraction from cocoa byproducts.

### 3.3 Total Phenolics Extraction and Antioxidant Activity

Phenolic compounds, including phenolic acids and flavonoids, were quantified in the CBS extracts obtained under optimized conditions using the Folin–Ciocalteu method. For comparative purposes, an additional extraction was performed using 40% ethanol, a solvent commonly employed for the recovery of phenolics from plant matrices due to its polarity and compatibility with aqueous systems.^44^

The TPC obtained using the optimized NADES-based ultrasound-assisted extraction was 91.79 ± 3.77 mg GAE g^–1^ DM, representing a 14.6% increase compared to the value reported in our previous study using ethanol 40% and the EDGE system (80.10 ± 2.89 mg GAE g^–1^ DM).^12^ This improvement highlights the enhanced efficiency of NADES in extracting phenolic compounds from cocoa bean shells, likely due to their strong hydrogen bonding capacity and ability to stabilize polyphenolic structures.

The total phenolic content of the CBS extract obtained with NADES under optimized conditions was 1.17 times higher than that achieved with 40% ethanol, highlighting the superior extraction efficiency of the eutectic solvent system (Table 4). The integration of UAE with NADES has proven to be an effective strategy for extracting other phenolic compounds from CBS,^31^ as well as from byproducts of the olive oil, onion, tomato, and pear industries,^44^ while simultaneously reducing the reliance on organic solvents. For instance, Martinović et al.^45^ compared the extraction efficiency of natural deep eutectic solvents (NaDES) with that of conventional solvents such as ethanol and water in different plant matrices, including bilberry fruits, bilberry leaves, and green tea. The authors reported that NaDES—particularly the betaine–urea system—yielded significantly higher amounts of total phenolics and flavonoids. In agreement, NADES extracts produced by Lazović et al.^19^ had higher TPC values compared to methanol.

The DPPH and ABTS assays confirmed the hydrogen atom/electron-donating capacity, providing valuable insights into the intrinsic antioxidant potential of CBS extracts in vivo.^46^ The NADES extract, obtained under optimized conditions, demonstrated significantly enhanced antioxidant activity, with radical scavenging capacities 2.05-fold higher in the DPPH assay and 1.45-fold higher in the ABTS assay compared to 40% ethanol, confirming the superior performance of the eutectic system.

The superior antioxidant performance of NADES extracts observed in this study is consistent with previous findings in the literature. Martinović et al.^45^ demonstrated that NADES-based extracts from bilberry and green tea exhibited significantly higher antioxidant activity (DPPH, ABTS, FRAP) than those obtained with ethanol or water, particularly when using a betaine–urea solvent system. These results were attributed to the improved solubilization and stabilization of phenolic compounds within the NADES matrix, as well as the green and tunable nature of these solvents. Deep eutectic solvents (DES) can stabilize extracted solutes through strong molecular interactions, primarily hydrogen bonding between solutes and solvent components.^47^ These interactions reduce the solutes’ availability to react with atmospheric oxygen.^48,49^

### 3.4 Analytical Performance of HPLC Method

The proposed HPLC method was validated for the following parameters: linearity, limit of detection (LOD), limit of quantification (LOQ), precision, and accuracy (Table 5). Linearity was determined using the external standard method. The calibration curve was constructed with standard solutions at eight different concentrations (1–150 mg L^–1^) of theobromine and caffeine, measured in triplicate, with the peak area plotted against concentration. ANOVA at the 95% confidence level demonstrated that the regression was significant, and the linear regression coefficients (*R* ^2^) > 0.99 attested a good linearity of the method. The limit of detection (LOD) The limit of detection (LOD) and limit of quantification (LOQ) were determined from the calibration curve data, according to IUPAC.^26^

**Table 5:** Validation Parameters of the Developed HPLC/DAD Method

					precision^e^		
compound	regression equation^a^	*R* ^2^ ^b^	LOD (mg L^–1^)^c^	LOQ (mg L^–1^)^d^	intraday (*n* = 7)	interday (*n* = 7)	relative recovery^f^ (%) (*n* = 3)
theobromine	*y* = 19769*x* + 12,348	0.9992	2.97	9.01	1.10	0.26	98.35
caffeine	*y* = 28,808*x* + 6972	0.9997	1.37	4.17	1.63	0.62	119.42

a In the regression equation *y* = *ax* + *b*, *y* refers to the peak area, and *x* refers to the concentration of the analyte in mg L^–1^.

b *R* ^2^: square value of the correlation coefficient of the equation.

c Limit of detection.

d Limit of quantification.

e Average relative standard deviation for two different measurements (10 and 30 mg L^–1^), expressed as %.

f Average recoveries at two spike levels (10 and 30 mg L^–1^).

Precision was evaluated in terms of repeatability (intraday precision) and intermediate precision (interday precision) and expressed as the relative standard deviation (RSD). The RSD for all analytes was below 2.0%. The average accuracy results are shown in Table 5. Recoveries were performed by adding the standard to the sample, and the concentration was determined using the proposed method. The recovery percentage was calculated using the eq 3.

Relative recovery (RR, %) in the cocoa-shell matrix ranged from 87.4–109.2% for theobromine and 113.4–125.3% for caffeine (Table 5). Bias versus 100% recovery was evaluated by a one-sample, two-tailed *t*-test of RR (α = 0.05; *n* = 2; df = 1; *t*_crit_ = 12.71). For theobromine, L1 was 83.6 ± 1.8% (*t* = 13.16; significantly below 100%) and L2 104.4 ± 2.6% (*t* = 2.37; not significantly different). For caffeine, L1 was 96.6 ± 27.4% (*t* = 0.18; not significantly different) and L2 128.5 ± 0.6% (*t* = 63.00; significantly above 100%), consistent with the matrix effect below. Matrix effect was evaluated by comparing the slope of a matrix-matched calibration (Δarea vs added concentration in spiked extracts) with the solvent-based calibration, using ME% = (*m*_matrix_/*m*_solvent_ – 1) × 100. Theobromine showed −0.10% (negligible), whereas caffeine showed +26.5% (moderate enhancement), consistent with RR > 100%. In this study we quantified by external calibration; for applications requiring highest accuracy, we recommend matrix-matched or standard-addition calibration and/or modest extract dilution to compensate for this enhancement.

### 3.5 Greenness Assessment of Extraction Approach

To evaluate the environmental impact of analytical methods, several tools have been developed in alignment with the principles of Green Analytical Chemistry (GAC). One of the most comprehensive is AGREE, introduced by Pena-Pereira et al.,^27^ which assesses the greenness of analytical procedures based on the 12 principles of GAC. These principles emphasize the reduction of environmental impact and improvement of analytical sustainability through strategies such as minimizing sample size and waste generation, avoiding derivatization, and reducing energy and reagent consumption. They also encourage the use of direct and in situ techniques, automation, miniaturization, and multiparameter approaches. Additionally, the selection of nontoxic, renewable reagents and ensuring operator safety are integral to these guidelines. The 12 principles address: (1) direct techniques; (2) minimal sample size; (3) in situ measurements; (4) process integration; (5) automation and miniaturization; (6) avoidance of derivatization; (7) waste minimization; (8) multianalyte capability; (9) energy efficiency; (10) renewable reagents; (11) nontoxic reagents; and (12) operator safety. Together, these principles guide the development of safer, more efficient, and environmentally responsible analytical practices. AGREE evaluates how well a method aligns with these principles and generates a final greenness score ranging from 0 (not green) to 1 (fully green).

To complement the AGREE tool and focus specifically on the sample preparation step, AGREEprep was developed.^28^ This tool is designed to assess adherence to the ten principles of green sample preparation, including the use of in situ techniques, safer solvents and reagents, and renewable materials. It encourages minimizing waste and reducing the amounts of samples, chemicals, and materials used, while maximizing throughput and efficiency. The tool also prioritizes the integration of steps, automation, and low energy consumption to streamline processes and reduce environmental impact. Additionally, AGREEprep guides the selection of greener postpreparation analytical configurations and emphasizes operator safety through the evaluation of procedures and materials. These ten principles are (1) favor in situ sample preparation; (2) use safer solvents and reagents; (3) utilize renewable and reusable materials; (4) minimize waste; (5) reduce sample and chemical consumption; (6) maximize throughput; (7) integrate and automate processes; (8) minimize energy consumption; (9) adopt green analytical configurations; and (10) ensure operator safety. This framework supports the development of sample preparation methods that are both effective and environmentally responsible.

Table 6 presents the pictogram generated by the AGREE and AGREEprep metric tools. In this study, the AGREE score for the full analytical procedure was 0.55, indicating a moderate degree of greenness. Strong alignment was observed with principles related to minimal sample size (Principle 2), integration of steps (Principle 4), and the avoidance of derivatization (Principle 6). The use of a low-energy extraction method and safe NADES also contributed positively to Principle 9, which promotes the reduction of energy consumption during all analytical steps, and Principle 12, which ensures operator safety through the use of safe materials and procedures. However, the use of acetonitrile in the HPLC mobile phase negatively impacted the performance on Principle 11 (avoidance of toxic reagents). Although acetonitrile is widely employed in HPLC methodologies for the extraction and analysis of phenolic compounds and methylxanthines 7,8,12, its toxicity and nonrenewable origin reduce the overall greenness of the method. Additionally, the absence of in situ analysis contributed to lower scores for Principle 1, which promotes the use of direct analytical techniques to avoid sample preparation, and Principle 3, which encourages in situ measurements to minimize sample handling, transport, and associated environmental burdens.

**Table 6:**
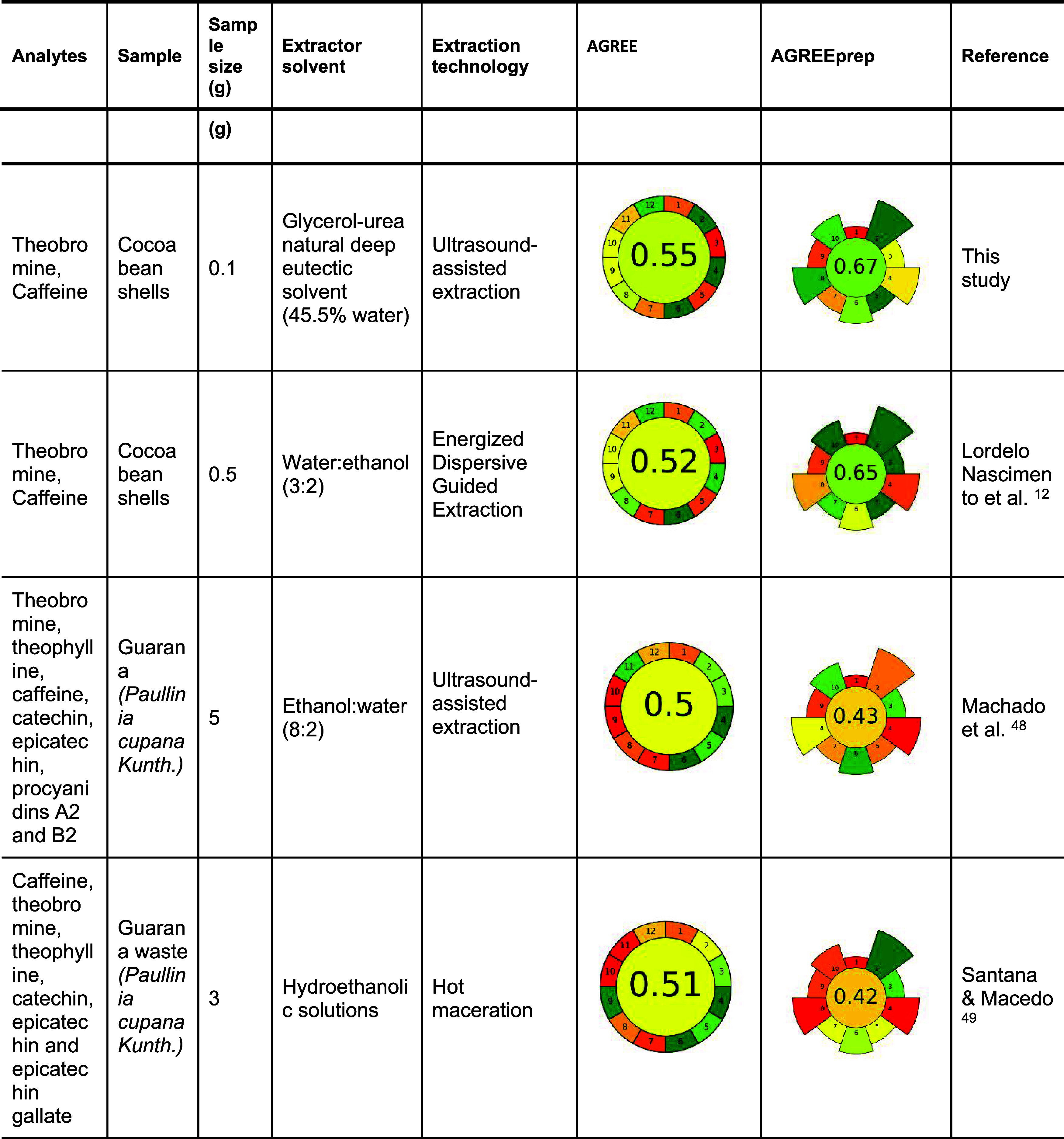
Greenness Assessment of Different Extraction Methods Based on AGREE and AGREEprep Metric Tools^48,49^

The AGREEprep score, which evaluates the environmental performance of the sample preparation stage, was higher at 0.67, indicating a greener and more sustainable protocol. The method showed strong alignment with several core principles of green sample preparation. It fully complied with Principle 2, by employing a nontoxic, biodegradable NADES composed of glycerol and urea, thus eliminating hazardous solvents. The small quantities of sample (100 mg) and solvent (3 mL) used supported Principle 5, which encourages the minimization of sample and chemical inputs. A short extraction time of 4 min enabled rapid processing, contributing positively to Principle 6 on maximizing sample throughput. The use of low-power ultrasound equipment and brief processing times aligned well with Principle 8, which aims to minimize energy consumption. Additionally, the method supported Principle 10, as the use of nonhazardous chemicals and simple handling procedures ensured safe conditions for the operator.

Moderate compliance was observed for other principles. The method partially adhered to Principle 3, as it employed sustainable and renewable materials but did not include solvent reuse, resulting in a single-use system. While NADES are derived from renewable sources, their one-time application limits the greenness of the protocol in this respect. Similarly, Principle 4, which encourages waste minimization, was addressed through low material use, but the inability to recycle reagents or materials reduced its overall performance. With respect to Principle 7, the sample preparation involved simple and sequential operations, achieving some level of process integration; however, it was not fully automated, requiring manual steps such as pipetting and tube handling.

In contrast, two principles were not fulfilled. The method did not comply with Principle 1, which favors in situ sample preparation to eliminate the need for sample transport and laboratory processing, nor with Principle 9, which emphasizes the selection of the greenest possible analytical configuration after sample preparation. In this case, the use of acetonitrile in the HPLC analysis, although common, is considered less green due to its toxicity and nonrenewable origin.

When compared to a previously developed method for methylxanthine extraction from cocoa bean shell using the Energized Dispersive Guided Extraction (EDGE) system with 40% ethanol,^12^ the current UAE-NADES approach exhibited superior environmental and analytical performance. These findings reinforce the value of integrating green solvents and extraction strategies within analytical workflows, contributing to the development of methods that are both effective and environmentally responsible.^29^

### 3.6 Perspectives: Scale-Up and Sustainability

Translation of the present UAE protocol to larger throughputs is feasible using recirculating/flow-through ultrasonic reactors, which are widely described for fluid–solid extractions. Key requirements are maintaining comparable acoustic energy density and cavitation uniformity, while managing slurry viscosity and solids loading. Continuous and pilot-scale implementations have been reported for plant matrices using tubular or multitransducer flow cells, supporting the practicality of flow operation. In our context, modest water addition and moderate temperature can lower NADES viscosity, improving pumping, heat removal, and filtration without unduly disrupting solvent structure—parameters that can be tuned to match the residence time used in our desirability-optimized conditions. Practical measures include particle-size standardization, prewetting, loop recirculation with inline temperature control, and staged solid–liquid separation.^50–53^

For food or nutraceutical uses, residual solvent must be minimized and verified for the intended product and jurisdiction. Notably, the individual components glycerin (glycerol) and urea are recognized by the United States Food and Drug Administration (FDA) as Generally recognized as safe (GRAS) when used in accordance with good manufacturing practice, providing a favorable context for application-readiness of a glycerol–urea NADES. Because glycerol–urea mixtures are essentially nonvolatile, removal relies on process steps other than evaporation, such as dilution/clarification, membrane or adsorption polishing, or back-extraction with food-grade ethanol–water, followed by residual-solvent specification and routine QC. From a sustainability perspective, the solvent components are low-cost and nonflammable, and the process reduces reliance on volatile organic solvents. Solvent stewardship—controlling makeup water to manage viscosity, implementing reuse across cycles with quality checks, and tracking simple green metrics (solvent-to-solid ratio, energy per batch, and E-factor)—can further improve environmental performance and document the method’s readiness for industrial adoption..^54^

## 4 Conclusions

This study demonstrates, for the first time, the feasibility of using glycerol–urea-based natural deep eutectic solvents in combination with ultrasound-assisted extraction for the recovery of methylxanthines from cocoa bean shells—an abundant agro-industrial byproduct. The integration of these biodegradable, low-toxicity solvents with a rapid and energy-efficient extraction technique represents a novel and sustainable alternative to conventional methods that rely on volatile organic solvents. The adaptability of the NADES system, evidenced by the viscosity modulation through water addition, reinforces its potential for application in diverse sample matrices and compound classes. Using a Doehlert matrix, optimal conditions for theobromine and caffeine extraction were successfully identified, and the AGREEprep tool was employed to assess and confirm the method’s alignment with the principles of green sample preparation. The developed method offers several advantages over traditional extraction protocols, including reduced extraction time, low solvent consumption, high extraction efficiency, and the use of low-cost, food-grade materials. Furthermore, its compatibility with scalable technologies—such as flow-through ultrasound equipment and inline solid–liquid handling—underscores its potential for industrial application. When coupled with simple filtration steps, the process enables the generation of enriched ingredient streams suitable for food or nutraceutical use. In summary, this work introduces a novel, fast, and environmentally responsible protocol for methylxanthine extraction, contributing to the advancement of green analytical chemistry and the valorization of food industry residues.
